# The Complexity of the Influence of Growth Substances, Heavy Metals, and Their Combination on the Volume Dynamics of Vacuoles Isolated from Red Beet (*Beta vulgaris* L.) Taproot Cells

**DOI:** 10.3390/ijms251910842

**Published:** 2024-10-09

**Authors:** Waldemar Karcz, Zbigniew Burdach

**Affiliations:** Institute of Biology, Biotechnology and Environmental Protection, Faculty of Natural Sciences, University of Silesia in Katowice, 40-032 Katowice, Poland; zbigniew.burdach@us.edu.pl

**Keywords:** *Beta vulgaris* L., IAA (indole-3-acetic acid), FC (fusicoccin), heavy metals, vacuole volume

## Abstract

The plant vacuole is a very dynamic organelle that can occupy more than 90% of the cell volume and is essential to plant cell growth and development, the processes in which auxin (indole-3-acetic acid, IAA) is a central player. It was found that when IAA or FC (fusicoccin) was present in the control medium of vacuoles isolated from red beet taproots at a final concentration of 1 µM, it increased their volume to a level that was 26% or 36% higher than that observed in the control medium without growth regulators, respectively. In the presence of IAA and FC, the time after which most vacuoles ruptured was about 10 min longer for IAA than for FC. However, when cadmium (Cd) or lead (Pb) was present in the control medium at a final concentration of 100 µM, it increased the volume of the vacuoles by about 26% or 80% compared to the control, respectively. The time after which the vacuoles ruptured was similar for both metals. The combined effect of IAA and Pb on the volume of the vacuoles was comparable with that observed in the presence of Pb only, while for FC combined with Pb, it was additive. The use of IAA or FC together with Cd caused in both cases a decrease in the vacuole volumes by about 50%. The data presented in this study are discussed, taking into account the structure and function of the vacuolar membrane (tonoplast) and their changes in the presence of growth substances, heavy metals, and their combination.

## 1. Introduction

The relationships between membrane transport across the plasmalemma and tonoplast, turgor pressure, vacuole volume dynamics, and plant cell expansion are complex and require elucidation. It is still unclear what role auxin (indole-3-acetic acid, IAA) plays in them. Understanding the relationships between these processes is important for explaining the molecular mechanism of plant cell growth and its regulation under conditions of biotic and abiotic stresses. One of the links in the above-mentioned relationships is the vacuole volume dynamics, which is a direct consequence of the water and solute transport across the tonoplast as well as the elastic properties of this membrane. To shed light on this phenomenon, experiments were performed in which the effect of growth substances, heavy metals, and their combination on vacuole volume dynamics were studied. Currently, the specific effect of the growth substances and heavy metals on the dynamics of vacuole volume is not known.

While it is well established that turgor pressure participates in cell growth expansion [1], it is still unclear what the signalling is between the vacuole and the processes that occur during cell growth expansion. Considering the effect of auxin on the ion transport across the plasma membrane, it should be noted that this effect is fairly well understood in cells of *Zea mays* coleoptiles. According to the so-called “acid growth theory”, auxin activates the PM H^+^-ATPase, which acidifies the apoplast and causes the activation of the enzymes that are involved in cell wall loosening reviewed in [2]. It is also well established that auxin-induced growth involves K^+^ uptake through voltage-dependent, inwardly rectifying K^+^ channels (ZMK1, *Zea mays* K^+^ channel 1), the activity of which contributes to water uptake across the plasma membrane and consequently to cell expansion [3,4]. It has also been shown that apart from the post-translational, auxin-dependent up-regulation of the K^+^ uptake channels, auxin also regulates the expression of the maize K^+^ uptake channel gene *ZMK1* [4].

Significantly less is known about the role of the vacuolar membrane, the tonoplast, in the auxin-mediated growth of plant cells. It was shown that K^+^ current across the tonoplast is primarily mediated by two main classes of tonoplast non-selective cation channels (NSCCs): slow-activating (SV) and fast-activating (FV) vacuolar channels. These channels differ in their selectivity, kinetics, and activation factors [5,6]. Recently, using the patch-clamp technique, it was shown [7] that in symmetrical 100 mM KCl (the same concentration of KCl on both sides of the tonoplast), when IAA was added to bath solution at a final concentration of 1 µM, it stimulated the slow-activating vacuolar (SV) channels (an increase in K^+^ current from outside into the vacuole) in red beet taproot vacuoles. This effect resulted from faster channel activation, an increased amplitude and number of opened SV channels. Recently, Burdach et al. [8] also showed that IAA regulates the activity of the fast-activating vacuolar (FV) channels, thereby causing changes in the K^+^ fluxes across the vacuolar membrane. The addition of IAA to the bath solution of red beet vacuoles significantly increased the amplitude of the single FV channel outward currents and the number of open channels. However, luminal auxin (auxin introduced into the vacuole using patch-clamp pipette) reduced both the outward and inward FV currents [8].

Vacuoles are very dynamic organelles whose morphology changes during plant growth and development [9,10]. Recently, it has been shown that auxin altered the appearance of the vacuoles in the root epidermal cells of *Arabidopsis thaliana* so that they became smaller and that auxin also inhibited the growth of the root epidermal cells [11]. It has been suggested [11] that auxin coordinates extracellular and intracellular components, such as cell wall acidification and vacuolar morphogenesis, for driving and restricting cellular growth. According to the authors [11], such a dual growth mechanism would allow plants to dynamically de- and accelerate cellular expansion. This finding was used by Dünser and Kleine-Vehn [12] to propose the “acid growth balloon theory” according to which plant growth is the interplay between the intracellular space-filling “vacuolar balloon” and the required extracellular cell wall acidification/loosening. For recent discoveries regarding the effect of auxin (IAA) on vacuolar morphology and the contribution of plant vacuoles to cell growth, see [13,14].

It should also be added that auxin (IAA) and its metabolites are present in plant vacuoles and that auxin transport across the tonoplast plays an essential role in maintaining auxin homeostasis [15]. Taking into account the fact that plant vacuoles are highly dynamic organelles and are essential for growth and development, we performed experiments in which the effects of growth substances (IAA and FC), heavy metals (Pb and Cd), and their combination on the volume changes of vacuoles isolated from red beet (*Beta vulgaris* L.) taproot cells were studied. In this way, we tried to understand the mechanisms underlying the interaction of tonoplasts with growth substances and heavy metals, as well as their role in the regulation of vacuole volume dynamics, which is crucial for plant cell expansion.

To the best of our knowledge, no research has been reported in the literature (excluding our previous experiments with IAA [7]) on the effects of the above-mentioned substances on the post-mitotic vacuole volume regulation.

## 2. Results

### 2.1. Effect of IAA and FC on the Volume Changes of Red Beet Taproot Vacuoles

**Figure 1:**
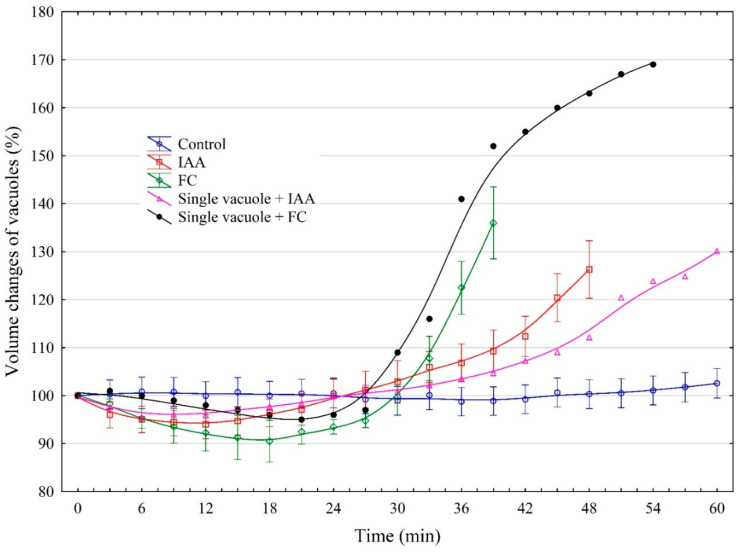
The effects of 1 μM indole-3-acetic acid (IAA) and fusicoccin (FC) on the volume changes of vacuoles isolated from red beet (*Beta vulgaris* L.) taproots. The vacuoles had been isolated directly onto glass slides by rinsing the surface of the fresh tissue slices with a control medium and a control medium with IAA or FC. The data points are the means of vacuole volumes (±SE) obtained from at least six single vacuoles. The volume of single vacuoles was calculated from the diameter of the vacuoles in a photographic image. The volume of the vacuoles was measured at the indicated times and converted to a percentage of the initial value (fixed as 100%). For the experiments, vacuoles with diameters in the range of 20–40 µm were selected. This figure also shows the volume changes of individual vacuoles that burst after a time longer than the vacuoles taken to calculate the mean values.

Figure 1 shows the volume changes of red beet taproot vacuoles incubated in the control medium and in the presence of both auxin (indole-3-acetic acid, IAA) and fusicoccin (FC). As Figure 1 indicates, the volume of the vacuoles that were incubated in the control medium practically did not change over 60 min. It should also be added here that the diameter of the vacuoles measured at 0 time is not the critical parameter that determines the time (at least over the first 60 min) after which the vacuoles are disrupted. However, when the vacuoles were incubated in the control medium with 1 µM IAA, their volume changed in a characteristic manner; the initial, transient decrease by, ca., 5% was followed by an increase during which the volume of the vacuoles was about 26.4% greater than the original value (100% at 0 time). Usually after 48 min, the vacuoles ruptured, although at least one in four ruptured later (Figure 1).

However, when the vacuoles were incubated in the presence of 1 µM FC, their volume, after a transient decrease by, ca., 10%, increased up to 136% (36% greater than the original value, 100% at 0 time) at 39 min, whereupon most of the vacuoles (at least four out of five) ruptured. In the presence of IAA, the time after which the vacuoles ruptured was, ca., 10 min longer than that for FC. Figure 1 also shows two examples of single vacuoles, which, during incubation with IAA or FC, ruptured significantly later than the vacuoles taken to calculate the mean values. For example, one in five vacuoles incubated in the presence of FC ruptured at 54 min, while one in four incubated in the presence of IAA ruptured at 60 min.

**Figure 2:**
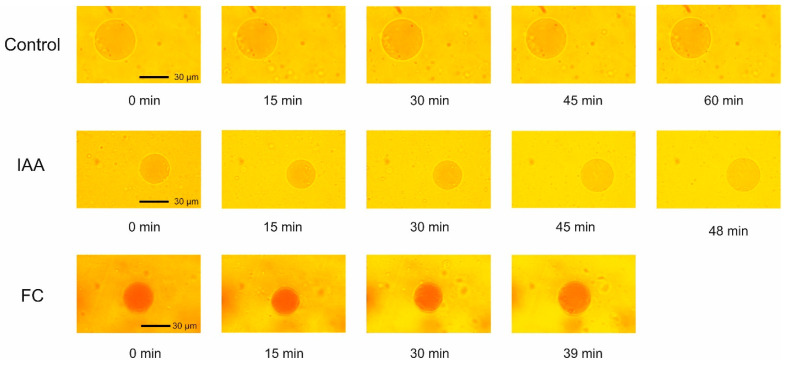
The photographs of the vacuoles incubated in the control medium and in the presence of IAA and FC. In order to show that the volume of the vacuole in the control medium practically does not change within 60 min, we showed a vacuole with a large diameter. The diameter of the vacuoles measured at 0 time is not the critical parameter that determines the time (at least over the first 60 min) after which the vacuoles disrupted.

Figure 2 shows the photographs of the vacuoles incubated in the presence of IAA and FC. They reflect the volume changes of the vacuoles and the time after which most of the vacuoles ruptured.

As the photographs indicate, in the presence of IAA, the time after which the most vacuoles ruptured was, ca., 10 min longer than that for FC.

### 2.2. The Effect of IAA and FC and Their Combination with Pb on the Vacuole Volume Dynamics

**Figure 3:**
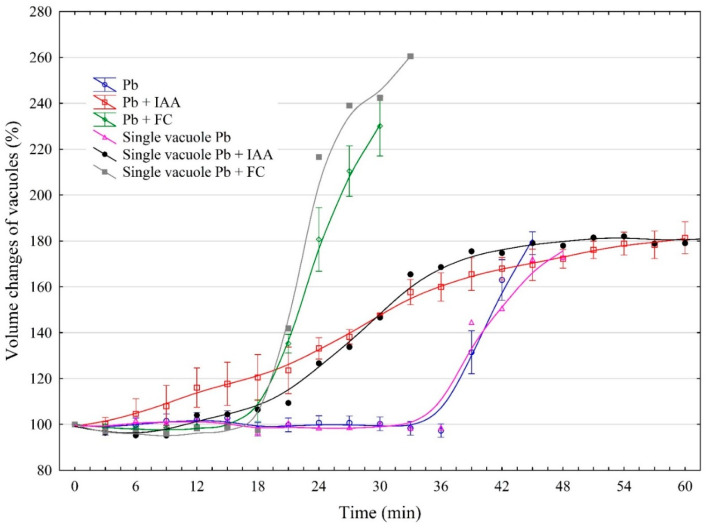
The effects of 1 μM indole-3-acetic acid (IAA) or 1 μM fusicoccin (FC) applied together with Pb (100 µM) on the volume changes of vacuoles isolated from red beet (*Beta vulgaris* L.) taproots. The vacuoles had been isolated directly onto glass slides by rinsing the surface of the fresh tissue slices with a bathing medium. The data points are the means (±SE) from at least six independent measurements performed every 3 min for the diameter of a single vacuole.

The volume of the vacuoles that were incubated in the control medium with 100 µM Pb practically did not change over the first 36 min, whereupon it dramatically increased up to 180% within the next 12 min (Figure 3). After this time, most vacuoles ruptured, although there were cases when the vacuoles ruptured after a longer time.

When the vacuoles were incubated together with IAA and Pb, their volume gradually increased up to 180%, over 60 min. However, in the presence of both FC and Pb, the volume of the vacuoles increased dramatically up to 230% with time intervals between 18 and 30 min, while in the first 18 min, the volume of the vacuoles did not change significantly. Interestingly, when the vacuoles were incubated together with FC and Pb, they usually ruptured after 30 min, which was half the time for IAA and Pb added simultaneously. In the presence of Pb alone, the vacuoles ruptured after 45 min. When IAA was added together with Pb, their combined effect was comparable with that observed in the presence of Pb only; however, when FC and Pb were present together, their effect was additive. It should also be added that IAA in contrast to FC significantly increases the time after which the vacuoles burst.

**Figure 4:**
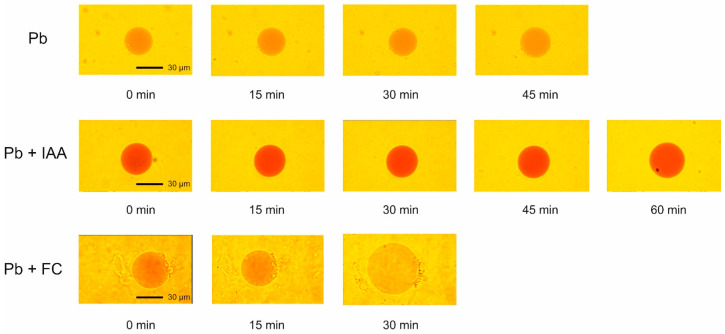
The photographs of the vacuoles incubated in the control medium with Pb and in the presence of IAA or FC combined with Pb.

Figure 4 shows the photographs of the vacuoles incubated in the presence of Pb and Pb added together with IAA or FC. They reflect the volume changes of the vacuoles and the time after which most of the vacuoles ruptured.

As the photographs indicate, in the presence of IAA added together with Pb, the time after which the most vacuoles ruptured was 2-fold longer than that for FC added together with Pb.

### 2.3. The Effect of IAA and FC and Their Combination with Cd on the Volume Changes in Vacuoles

**Figure 5:**
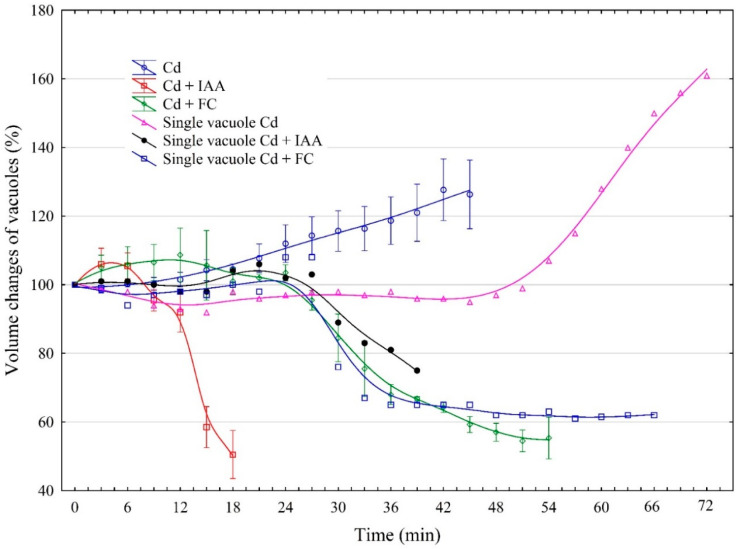
The effects of 1 μM indole-3-acetic acid (IAA) or 1 μM fusicoccin (FC) applied together with Cd (100 µM) on the volume changes of vacuoles isolated from red beet (*Beta vulgaris* L.) taproots. The vacuoles had been isolated directly onto glass slides by rinsing the surface of the fresh tissue slices with a bathing medium. The data points are the means (±SE) from at least six independent measurements performed every 3 min for the diameter of a single vacuole.

The addition of Cd at a final concentration of 100 µM (Figure 5) increased the volume of the vacuoles by, ca., 26% over 45 min. After this time, most vacuoles ruptured, although there was a case where the vacuole ruptured after 72 min (Figure 5). In this case, the volume of the vacuole started to increase after, ca., 54 min and reached a level that was 60% higher than that observed in the control medium. However, when IAA and Cd were added together, their effect differed from that when the substances were applied separately, and in this case, a rapid decrease in the vacuole volumes by about 50% was observed. Similarly to IAA, FC applied together with Cd decreased the volume of the vacuoles to a level comparable to that observed in the presence of IAA. Interestingly, the time after which the vacuoles ruptured was significantly shorter for vacuoles incubated in the presence of IAA and Cd added together than in the variant where FC was added together with Cd.

**Figure 6:**
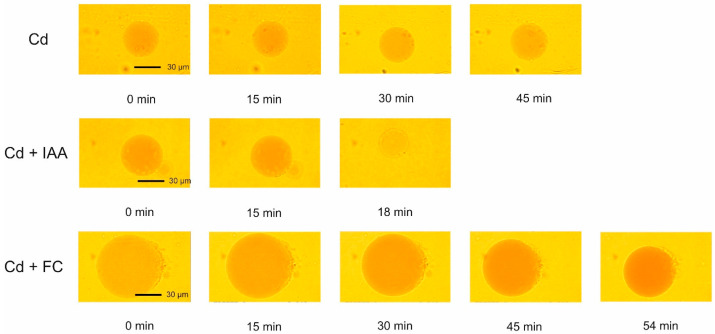
The photographs of the vacuoles incubated in the control medium with Cd and in the presence of growth substances (IAA and FC) combined with Cd.

Figure 6 shows the photographs of the vacuoles incubated in the presence of Cd and Cd added together with IAA or FC. They reflect the volume changes of the vacuoles and the time after which the most of vacuoles ruptured.

As the photographs indicate, in the presence of IAA added together with Cd, the time after which the most vacuoles ruptured was 3-fold shorter than that for FC added together with Cd.

### 2.4. A Comparison of the Effects of Growth Substances and Heavy Metals as Well as Their Combination on Both the Volume Changes of Vacuoles and the Time after Which the Vacuoles Ruptured

**Figure 7:**
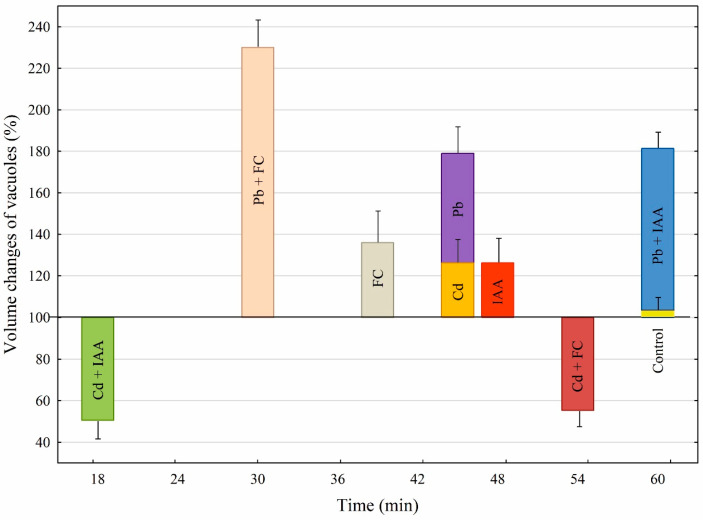
A comparison of the effects of growth substances (IAA and FC), heavy metals (Pb and Cd), and their combination on the maximal volume changes of vacuole immediately prior to their rupture. Moreover, these changes are also shown as a function of time after which the vacuoles ruptured. Bars indicate means ± SE taken from Figure 1, Figure 3 and Figure 5.

Figure 7 compares the volume changes of the vacuoles incubated in the presence of growth substances (IAA and FC) and heavy metals (Pb and Cd), as well as their combination. In addition, Figure 7 also shows the times after which the vacuoles ruptured at different variants of the experiments.

The data that are summarized in Figure 7 show that when IAA or FC was added to the control medium, it increased the volume of the vacuoles to the level that was 26.4% or 36% higher than that observed in the control medium, respectively. In the presence of IAA, the time after which the vacuoles ruptured was, ca., 10 min longer than that for FC. When Cd or Pb was added to the control medium, it increased the volume of the vacuoles by, ca., 26% or 80% compared to the control (100% at 0 time), respectively. Interestingly, although, in the presence of Pb, the increase in the volume of the vacuoles was about 3-fold greater than for Cd, the time after which the vacuoles ruptured was similar for both metals. When IAA was added together with Pb, their combined effect was comparable with that observed in the presence of Pb only; however, when FC and Pb were present together, their effect was additive. The addition of IAA together with Cd caused a decrease in the vacuole volumes by, ca., 50%. A similar effect was observed in the presence of FC added together with Cd. The time after which the vacuoles ruptured was significantly shorter (3 times) for vacuoles incubated in the presence of IAA combined with Cd than for FC combined with Cd. Interestingly, in the case of Pb, auxin (IAA) prolongs the time after which the vacuoles burst, while in the case of Cd, IAA significantly shortens it.

## 3. Discussion

It is well known that cell division and cell elongation are fundamental processes for plant growth and development. Plant cells contain a large central vacuole that occupies up to 90% of the cell volume in many mature cells. It is well established that plant cell elongation is driven by a combination of the osmotic uptake of water in the vacuoles and altered cell wall relaxation. Both processes are fundamental for cell elongation [16,17]. Although cell wall relaxation and the role of auxin in it have been fairly well understood [18], our knowledge of the mechanisms of vacuole expansion is limited. Therefore, the question arises about what the role of auxin is in the process of vacuole expansion and how this role changes in the presence of abiotic stresses (here, Pb and Cd). This last question is also particularly important because the answer to it may provide new insights into the mechanisms of plant tolerance to heavy metals.

The results presented in this paper are considered in three aspects: first, the vacuole volume dynamics in the control medium (iso-osmotic solution) and in the presence of growth substances, auxin (IAA) and fusicoccin (FC) (Figure 1 and Figure 7). For our study, we chose FC, the phytotoxin produced by the fungus *Fusicoccin amygdali* Del., because it mimics the effects of auxin in many respects, although its mode of action at the molecular level differs from that of auxin [19,20,21,22]. The experiments presented here clearly show that practically no volume changes were observed when selected vacuoles were placed in the iso-osmotic solution (Figure 1). In accordance with the data obtained by [23], the volume of the beetroot vacuoles incubated in the iso-osmotic solution depends on the balance between the water and solute permeabilities. The authors also showed that beetroot vacuoles can hold its steady state in the pH range between 6.0 and 8.5, which is also confirmed by our experiments (here pH 7.5). However, when IAA or FC was present in the control medium, it increased the volume of the vacuoles to a level that was 26.4% or 36% higher than that observed in the control medium, respectively. Interestingly, in the presence of IAA, the time after which most of the vacuoles ruptured was, ca., 10 min longer than that for FC (Figure 1 and Figure 7).

To shed light on this phenomenon, we compared our results with those obtained by [24] in experiments performed on isolated protoplasts as well as on cells of maize coleoptile segments. These authors showed that, immediately after the addition of IAA, at a final concentration of 1 µM, to the incubation medium of maize coleoptile protoplasts, their transient shrinkage was observed, followed by a long-term (more than 60 min) swelling response. A similar, but slightly faster and greater effect was observed by the authors for 1 µM FC. In addition, the authors also showed that the IAA- and FC-induced volume changes of protoplasts occurred in the same time window as the growth of maize coleoptile cells induced by these substances. It should be stated here that our findings with the vacuoles isolated from red beet (*Beta vulgaris* L.) taproots are qualitatively similar to that obtained by [24], suggesting that protoplast volume changes may be dominated by vacuole volume dynamics.

Trying to explain the mechanism of the IAA- and FC-induced increase in the volume of vacuoles isolated from red beet taproots, the following conditions should be taken into account; 1: the composition of the medium in which the vacuoles were incubated; 2: the water and solute transport across the tonoplast; and 3: the membrane (tonoplast) tension. As indicated in the Materials and Methods section, the incubation medium contained only mannitol (650 mM) and HEPES; pH 7.5. As was previously shown by [25], the transport of mannitol across the tonoplast of red beet vacuoles is passive. The passive nature of mannitol transport was also obtained by [26] in experiments performed with the vacuoles isolated from storage parenchyma of celery petioles. It should also be added that the membrane potential of the vacuoles incubated in the presence of sorbitol (550 mM) is low (8.8 mV) ([27]; see also [28]).

The increase in the vacuole volume observed in the presence of IAA or FC is a consequence of water influx into the vacuole as a result of osmotic and hydrostatic forces. It is worth mentioning here the research conducted on red beet (*Beta vulgaris*) vacuoles by [29]. The authors in that study simultaneously measured the mechanical behaviour and the water transport rates during the osmotic response of emptied-out oocytes expressing *Bv*TIP1;2 (TIP type-vacuole 1 aquaporin identified from the tonoplast of vacuoles from red beet cells), showing that this water channel is a mechanosensitive member of the aquaporin family and, similarly to grapevine tonoplast aquaporin TIP2;1 [30], is regulated by membrane tension changes (changing from an open to a closed state in an internal-vacuolar-pressure-dependent manner). In the scientific literature, it is possible to find data concerning the effect of natural (IAA) and synthetic (NAA) auxin on the organization of plant and animal model membranes [31]. The authors showed that both auxins decrease the thermodynamic stability of lipid monolayers and their condensation, as well as weaken the interactions between monolayer components. They also found that the shape of the surface pressure–area isotherms for the monolayers imitating *Arabidopsis thaliana* and rat liver membranes, formed on auxins solutions, is characterized by the decrease in the surface pressure during the decrease in monolayer condensation. Taking into account the above, a hypothesis can be formulated according to which the increase in vacuolar volume observed in the presence of auxin (also probably in the presence of fusicoccin) is caused by the auxin-induced decrease in tonoplast condensation (decrease in tonoplast membrane tension), which in turn causes the reduction in hydrostatic pressure in the vacuole and the opening of the water channels (*Bv*TIP1;2 aquaporin) in the vacuolar membrane (tonoplast). Because of this, water flows into the vacuoles and increases their volume.

The second aspect of our experiments concerns the effect of Pb and Cd on the volume changes of vacuoles (Figure 3 and Figure 7). It is generally accepted that both metals are very toxic elements for plants. Although the most common toxic symptom of these heavy metals in plants is reduced growth, the mechanism of this phenomenon is not fully understood [32]. Our findings showed that when Cd or Pb was added, it increased the volume of the vacuoles by, ca., 26% or 80% compared to the control medium, respectively. Interestingly, the time after which the vacuoles ruptured was similar for both metals. As far as we know, there are no data regarding the influence of Cd and Pb on tonoplast aquaporins, but there are data on the impact of heavy metals on the activity (or gating) of plasma membrane water channels (AQPs). For example, it was found that the conductivity of AQPs in the plasma membrane of *Allium cepa* cells is decreased in response to Cd, Hg, Pb, and Zn [33]. However, the experiments performed on lipid model membranes [34,35] showed that Pb and Cd affect the condensation and morphology of the lipid membranes, depending on the kind of metal used. The differences in the mechanism of both metal action on membranes is also confirmed by our studies in which we showed that Pb, at the same time as Cd, causes a three-times-greater increase in the volume of vacuoles compared to Cd.

The third aspect concerns the effect of growth substances and their combination with heavy metals on the volume changes of the vacuoles (Figure 5 and Figure 7). We found here that when IAA was added together with Pb, their combined effect was comparable with that observed in the presence of Pb only. However, the time after which the vacuoles ruptured was 15 min longer for IAA combined with Pb than for Pb only. In contrast to IAA, when FC and Pb were present together, their effect on the vacuole volume was additive, while the time after which the vacuoles ruptured was 30 min shorter. This observation may suggest a different mechanism of the action of both growth substances on membrane lipids. In the case of IAA added together with Pb, it is obvious that IAA weakens the effect of Pb. This result is in good agreement with the data obtained by Hąc-Wydro et al. [34] who showed that lead(II) ions and auxin (IAA) used separately change the properties of lipid model membranes; however, the mixing of metal ions with auxin makes the influence of sole metal weaker. Interestingly, the combined effect of Cd and growth substances on vacuole volume dynamics looks completely different than that for Pb. The addition of IAA or FC together with Cd caused a decrease in the vacuole volumes by, ca., 50%. The time after which the vacuoles ruptured was significantly shorter (three times) for vacuoles incubated in the presence of IAA combined with Cd than for FC mixed with Cd. As proposed by Hąc-Wydro et al. [35], the differences in the influence of sole metal ions and metal ions mixed with auxin on lipid monolayer morphology and organization results from the interactions occurring between metal cations and the carboxylic group of the auxin molecule. The verification of whether such a proposal may be valid for the vacuolar membrane requires further research.

How does the vacuole volume changes observed here relate to the plant cell growth? A partial answer to this question can be obtained by comparing the results presented here with the corresponding ones obtained for plant cells. In doing so, it is necessary to cite the experiments carried out with maize coleoptile cells (a classical model system for studies on plant cell elongation growth) treated with growth substance (IAA and FC), heavy metals (Pb and Cd), and their combination [36,37]. In these experiments, cadmium and lead, which were added at 100 µM, reduced the endogenous (growth in control medium) and IAA-induced elongation growth of maize coleoptile cells, while Cd was more effective in growth inhibition than Pb. In the case of FC, it was found [36] that FC combined with Cd counteracted the toxic effect of Cd on the endogenous growth of maize coleoptile cells.

However, it should be remembered that in the growth of a plant cell, in addition to the vacuole, the cell wall plays a decisive role. The question of whether the vacuole is a driver or a follower of cell growth is an open question [13]. The results presented here may suggest that the tested substances, after penetrating the cell, may directly affect the vacuolar membrane, causing the observed phenomena.

## 4. Materials and Methods

### 4.1. Plant Material

Storage roots of red beet (*Beta vulgaris* L.) of unknown variety were purchased from local supermarkets. The vacuoles were isolated using the nonenzymatic method that was previously described [38]. In this method, the intact vacuoles were mechanically isolated directly onto glass slides by rinsing the surface of fresh tissue slices with a bath solution containing 650 mM mannitol (Merck, Darmstadt, Germany) and 10 mM HEPES (Merck, Darmstadt, Germany), pH 7.5 (depicted as control medium), or with the control medium supplemented with growth substances (IAA or FC), heavy metals (Pb or Cd), and their combination. Vacuole diameters were measured at 60× magnification using an AX70 microscope (Olympus Provis) with fully automatic photomicrography that was connected to a Hamamatsu Colour Chilled 3CCD camera (C5810 model). The vacuoles with a diameter ranging from 20 to 40 µm were selected for the measurements.

### 4.2. Chemicals

Indole-3-acetic acid (IAA) (Serva, Heidelberg, Germany) was used as potassium salt since it could be rapidly dissolved in water. IAA at a final concentration of 1 µM was used. This IAA concentration was chosen in accordance with our previous experiments with red beet (*Beta vulgaris* L.) taproot vacuoles [7,8]. Fusicoccin (FC) (Sigma, USA) was dissolved in ethanol and added to the incubation medium at a final concentration of 1 µM. The maximal ethanol concentration of 0.2% did not affect the volume of the vacuoles. Cadmium (CdCl_2_ × 2.5 H_2_O) (Fluka, Buchs, Switzerland) and lead (PbCl_2_) (Fluka, Switzerland) were dissolved in deionised water and used at a final concentration of 100 µM. The concentration of both metals (100 µM) was selected in accordance with our previous studies [36,39,40], as well as by other authors [41,42,43], who showed that Cd and Pb at 100 µM caused a moderate toxic effect on plant growth.

### 4.3. Statistical Analysis

Data were analyzed with TIBCO Software Inc., Palo Alto, CA, USA (2017). Statistica (data analysis software system) version 13 (https://www.statsoft.pl/statistica_13/, accessed on 6 October 2024).

## Data Availability

The original contributions presented in this study are included in the article; further inquiries can be directed to the corresponding author.
